# *RUNX2* repeat variation does not drive craniofacial diversity in marsupials

**DOI:** 10.1186/s12862-017-0955-6

**Published:** 2017-05-04

**Authors:** Axel H. Newton, Charles Y. Feigin, Andrew J. Pask

**Affiliations:** The School of BioSciences, The University of Melbourne, Victoria, 3010 Australia

**Keywords:** Marsupial, Craniofacial, Evolution, RUNX2, CBFA1, Repeat variation, Repeat purity

## Abstract

**Background:**

Runt-related transcription factor 2 (*RUNX2*) is a transcription factor essential for skeletal development. Variation within the *RUNX2* polyglutamine / polyalanine (QA) repeat is correlated with facial length within orders of placental mammals and is suggested to be a major driver of craniofacial diversity. However, it is not known if this correlation exists outside of the placental mammals.

**Results:**

Here we examined the correlation between the RUNX2 QA repeat ratio and facial length in the naturally evolving sister group to the placental mammals, the marsupials. Marsupials have a diverse range of facial lengths similar to that seen in placental mammals. Despite their diversity there was almost no variation seen in the RUNX2 QA repeat across individuals spanning the entire marsupial infraclass. The extreme conservation of the marsupial RUNX2 QA repeat indicates it is under strong purifying selection. Despite this, we observed an unexpectedly high level of repeat purity.

**Conclusions:**

Unlike within orders of placental mammals, *RUNX2* repeat variation cannot drive craniofacial diversity in marsupials. We propose conservation of the marsupial RUNX2 QA repeat is driven by the constraint of accelerated ossification of the anterior skeleton to facilitate life in the pouch. Thus, marsupials must utilize alternate pathways to placental mammals to drive craniofacial evolution.

**Electronic supplementary material:**

The online version of this article (doi:10.1186/s12862-017-0955-6) contains supplementary material, which is available to authorized users.

## Background

Mammals have evolved a diverse array of craniofacial morphologies in response to their specialist diets. Examples of carnivores, omnivores and herbivores have evolved independently in both the placental (eutherian) and marsupial (metatherian) lineages [1–3] with diet placing substantial selective pressures on craniofacial evolution. While there are many key developmental genes responsible for patterning the facial skeleton, the precise mechanisms driving these morphological adaptations are not well defined [4]. One of the most remarkable examples of craniofacial diversity is between breeds of domestic dogs which have been subjected to strong artificial selection [5]. A comparative analysis of repeat variation in developmental genes associated with skeletal and craniofacial development was performed across dog breeds with diverse craniofacial phenotypes [6]. A strong positive correlation was observed between craniofacial length and the ratio of polyglutamines (Q) to polyalanines (A) in the QA repeat domain of the Runt-related transcription factor 2 (*RUNX2*) gene [6].

RUNX2 is a transcription factor and master regulator of osteogenesis in the development of the mammalian skeleton [7, 8]. RUNX2 binds the osteoblast-specific cis-acting element (OSE-2) within the promoter of several key skeletal genes [9]. Mice deficient for *Runx2* are unable to develop a bony skeleton [7]. In humans, mutations in *RUNX2* cause cleidocranial dysplasia (CCD), with individuals possessing craniofacial abnormalities with delayed closure of cranial sutures and dental anomalies [10]. Interestingly, several of the skeletal abnormalities associated with CCD patients are characteristic of the Neanderthal skeleton, and it has been hypothesized that evolutionary changes to *RUNX2* played a fundamental role in the phenotypic divergence of modern humans [11].

RUNX2 contains several functional domains, including a highly conserved RUNT DNA binding domain and, central to this study, a repetitive glutamine (Q), alanine (A) domain [12]. Changes to the length, or ratio, of sequential glutamines to alanines within RUNX2 alter its transactivational activity [13–17] providing a direct link between variation within this domain and craniofacial length in dogs. Amino acid coding repeats are thought to provide a fast-acting mechanism for generating evolvability. Repeats promote replication slippage, resulting in expansions and contractions which can generate new alleles in rapid succession and promote variation [18, 19]. The QA repeat in RUNX2 provides a mechanism by which rapid morphological variation can arise. Such protein coding repeats have been described as “evolutionary tuning knobs” where small, incremental changes can be associated with rapid morphological evolution [20, 21].

Selective pressures over short evolutionary timeframes may favour changes in repeat length as a mechanism to generate rapid morphological change [20, 21]. This may be especially true in the domestic dogs as they represent a single species that has been subjected to intense selective breeding over the past century [6]. The RUNX2 QA repeat and facial length was investigated across distinct placental orders to determine if repeat evolution correlates with facial morphology in naturally evolving taxa over larger evolutionary timeframes. The QA-repeat and facial-length was recorded across the carnivorans, the order in which the dogs and other canids are phylogenetically distributed [14]. As seen in the domestic dogs (a single species), there was a positive correlation between the RUNX2 QA repeat length and facial length within this order, despite over 40 million years of evolution [14]. It was also noted that this correlation was stronger in members of the Caniformia who exhibit allometric craniofacial development and higher overall craniofacial diversity. In addition, the RUNX2 repeat to facial length ratio was also examined across another placental order, the Primates, who have been evolving for 55 million years [22]. Primates, like the Carnivora, display a similar positive correlation between the RUNX2 repeat and facial length [23]. An analysis of the RUNX2 repeat to facial length was conducted across the three extant lineages of placental mammals including the Afrotheria, Boreoeutheria including Euarchontoglires (Primates, Rodents) and Laurasiatherians (Ungulates, Carnivora), and the Xenartha. While a correlation could be observed between the RUNX2 repeat and facial length within specific orders, this relationship did not hold true when comparing across orders spanning the entire placental infraclass [24].

Together these studies show a correlation between the *RUNX2* QA repeat and facial length within defined placental lineages. The modern orders of placental mammals arose ~65 million years ago [25] and radiated into a diverse range of terrestrial, aquatic and aerial species (for example, rodents, cetaceans and bats) with each group displaying highly variable facial morphologies acquired along their own evolutionary trajectories. Although the *RUNX2* repeat may drive facial evolution within orders, other compensatory epistatic changes may act across larger evolutionary distances such that correlations cannot be detected.

**Fig. 1:**
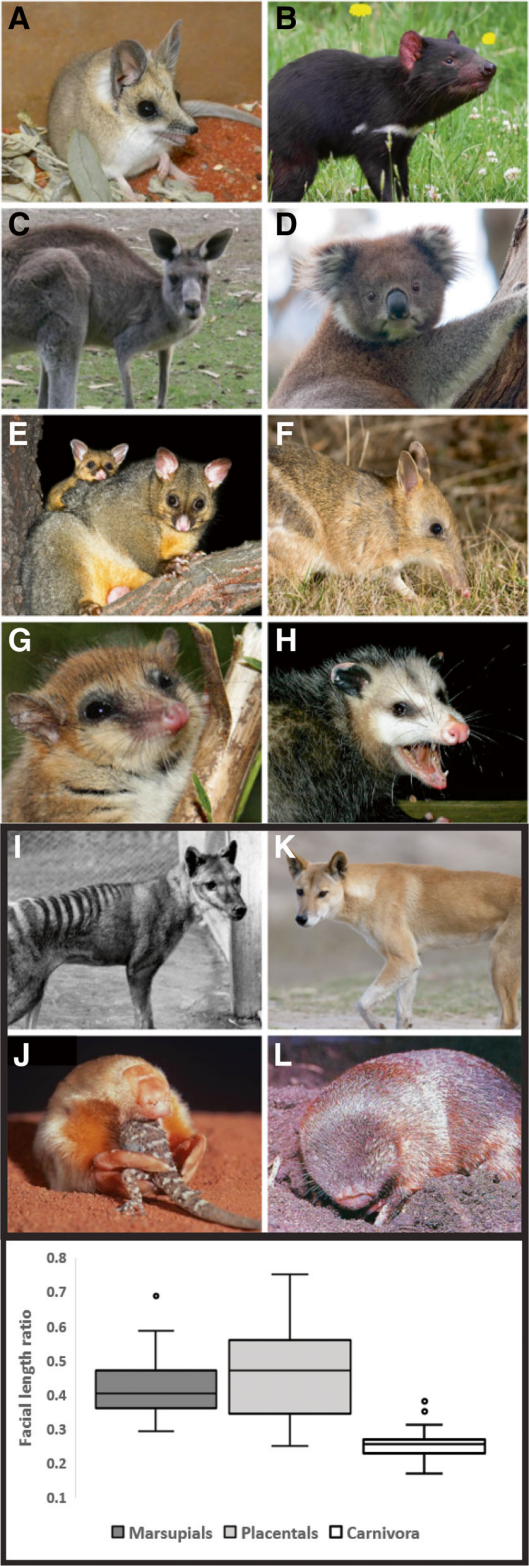
Craniofacial diversity of marsupials and convergenceMarsupials possess a diverse range of morphologies and craniofacial diversity. **a** Fat-tailed dunnart (*Sminthopsis crassicaudata*) and **b** Tasmanian devil (*Sarcophilus harrisii*) from the order Dasyuromorphia. **c** Eastern grey kangaroo (*Macropus giganteus*), **d** koala (*Phascolarctos cinereus*) and **e** brushtail possum (*Trichosurus vulpecula*) from the order Diprotodontia **f**. Eastern barred bandicoot (*Perameles gunnii*) from the order Peramelemorphia. **g** Monito del monte (*Dromiciops gliroides*) from the order Microbiotheria, and **h** Virginian opossum (*Didelphis virginiana*) from the order Didelphimorphia. Panels I-L marsupials and placentals that display striking craniofacial convergence. Thylacine (*Thylacinus cynocephalus*) **i** order Dasyuromorphia, and placental dingo (*Canis lupus dingo*) **k**; and the marsupial mole (*Notoryctes typhlops*) (J), order Notoryctemorphia and placental golden mole (*Chrysochloris sp.*) **l**. All images have been reproduced with permission (Additional file 4)(Additional file 1: Table S1 and Additional file 4: Table S4). **m** Box plot showing the range of marsupial facial length ratios compared with placentals [24] and Carnivora [14]. Boxes indicate the upper and lower quartiles, horizontal line inside indicates the median value. The horizontal lines indicate the extremes of the distribution with outliers shown as points

All studies on *RUNX2* repeat length and facial diversity have been conducted in placental mammals. However, it is unknown whether a correlation between *RUNX2* repeat length and facial length exists in their sister group, the marsupials. The marsupials are a naturally evolving infraclass that split from the placental lineage ~160 million years ago [26] and radiated into 7 orders distributed through two superorders (the Australidelphia and Ameridelphia). Like their placental relatives, marsupials have evolved their own distinct range of adaptive craniofacial morphologies suited to their various specialized feeding ecologies (Fig. 1a-j, [1–3]). Marsupials occupy a diverse range of facial lengths but show a reduced range of overall craniofacial diversity when compared to placental mammals (Fig. 1m; [1]). Within marsupials there are also several cases of convergent craniofacial phenotypes with the placentals (Fig. 1i-l). The extinct thylacine and the canids represent one of the most extraordinary examples of convergence in mammals especially in their craniofacial morphology (C.Y. Feigin, A.J. Pask personal communication). Here we test whether variation to the *RUNX2* repeat can explain facial length variation between individuals throughout the various orders and families of marsupials, and across the marsupial infraclass.

Surprisingly, in contrast to the placental mammals, marsupials possess almost no variability in the *RUNX2* repeat length or composition across individuals at the genus, family and order level. Despite their diverse array of facial morphologies, marsupials have not utilized evolutionary changes to the *RUNX2* repeat to control their facial length development.

## Results

### Marsupials display a wide range of craniofacial diversity

**Fig. 2:**
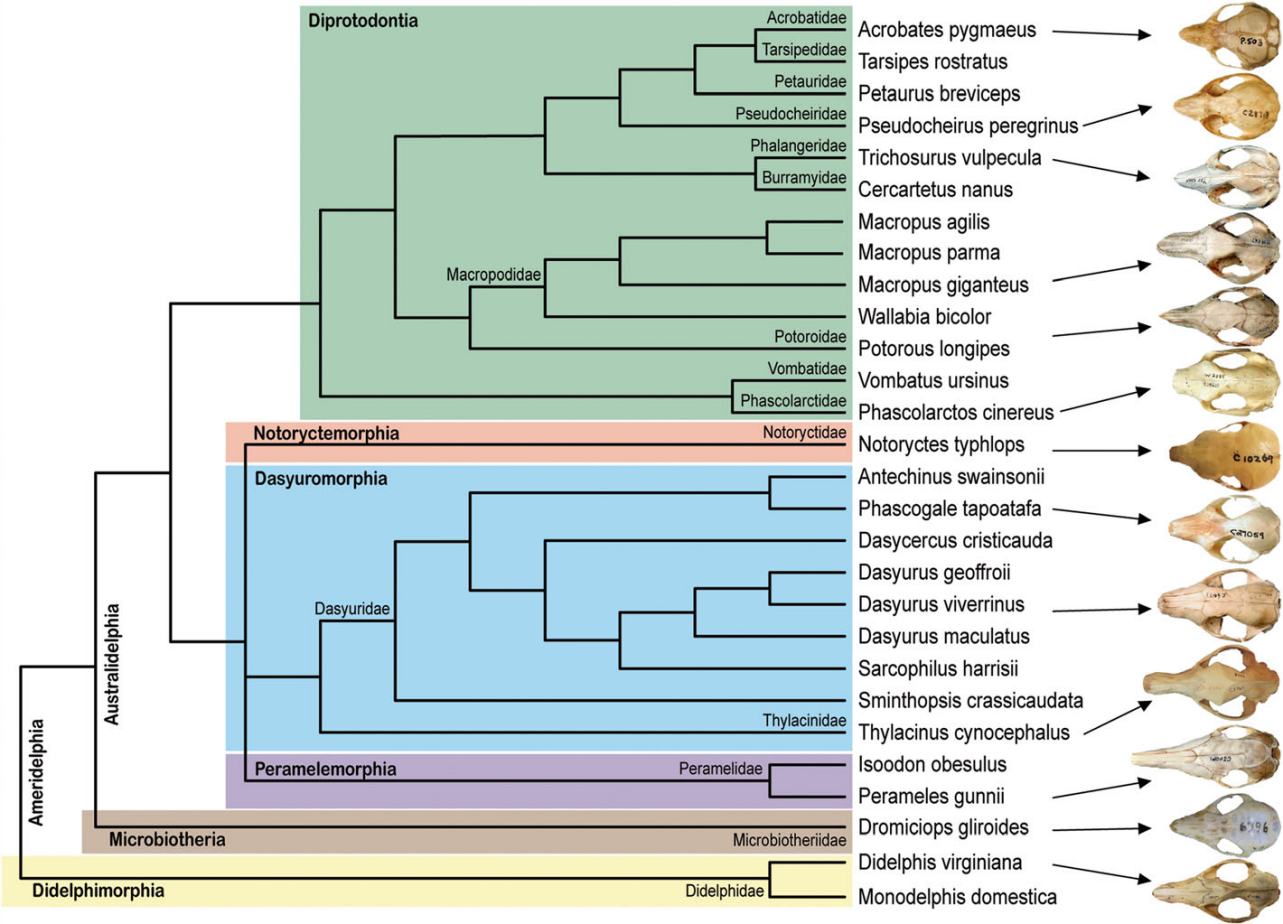
Phylogeny of sampled marsupials and craniofacial morphologyPhylogeny of marsupials used this study with selected taxa displaying a wide range of craniofacial diversity. Taxa included represent 6 of the 7 marsupial orders and 14 families. All skull images were taken from the Museum Victoria mammalogy collection with permission, with the exception of the *Dropiciops gliroides* (Adapted from [37]; Additional file 2: Table S2). Different colour lineage shading indicates different marsupial orders

**Fig. 3:**
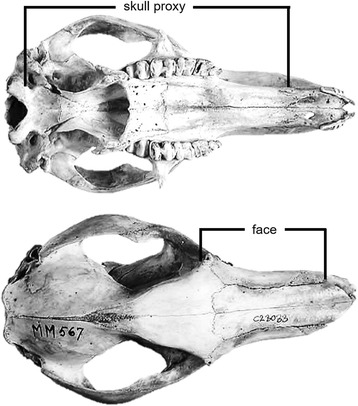
Facial length measurementsEastern Grey Kangaroo (*Macropus giganteus)* skull showing facial length measurements taken in the study. Size proxy was taken as skull length recorded from the lateral edge of the occipital condyle to the anterior juncture of the premaxilla and maxilla (*top panel*). Facial length was recorded as the distance between the junction of the lacrimal, jugal and maxillary bones to the junction of the nasal and premaxilla (*bottom panel*)

The marsupials display a wide range of adaptive craniofacial morphologies (Figs. 1 and 2), corresponding to their various diets. As expected, the marsupials display a diverse range of facial lengths, which are consistent with the range of facial length ratiosobserved within members of the placentals (Fig. 1m, [6, 14, 23, 24]). The majority of the marsupials displayed facial length ratios, measured as the length of the face divided by skull length (Fig. 3), between 0.32 and 0.54, including all of the Dasyuromorphia, Diprotodontia, Microbiotheria, and the Didelphimorphia. The koala, being a strict folivore, displayed the shortest facial length ratio of 0.293 while the omnivorous short-nosed Southern brown bandicoot and insectivorous marsupial mole displayed longer facial length ratios between 0.55–0.6. The major exception was seen in the long-nosed Eastern barred bandicoot, which possesses the longest face in this study with a facial length ratio of 0.69.

There are several cases of morphological convergence reported between marsupials and placental mammals with the most striking examples seen between the thylacine and canids (C.Y. Feigin, A.J. Pask personal communication), and the marsupial and placental moles [27]. We compared the facial length data from the thylacine to that reported for the canids [14], as well as between the marsupial mole (*Notoryctes typhlops*) and the African Cape Golden Mole (*Chrysochloris asiatica,* DigiMorph.org). As expected the thylacine and red fox displayed extremely similar facial length ratios (0.36 and 0.38 respectively). The marsupial mole and golden mole also showed similar ratios (0.58 and 0.51 respectively).

### Marsupials have a highly conserved *RUNX2* repeat

**Fig. 4:**
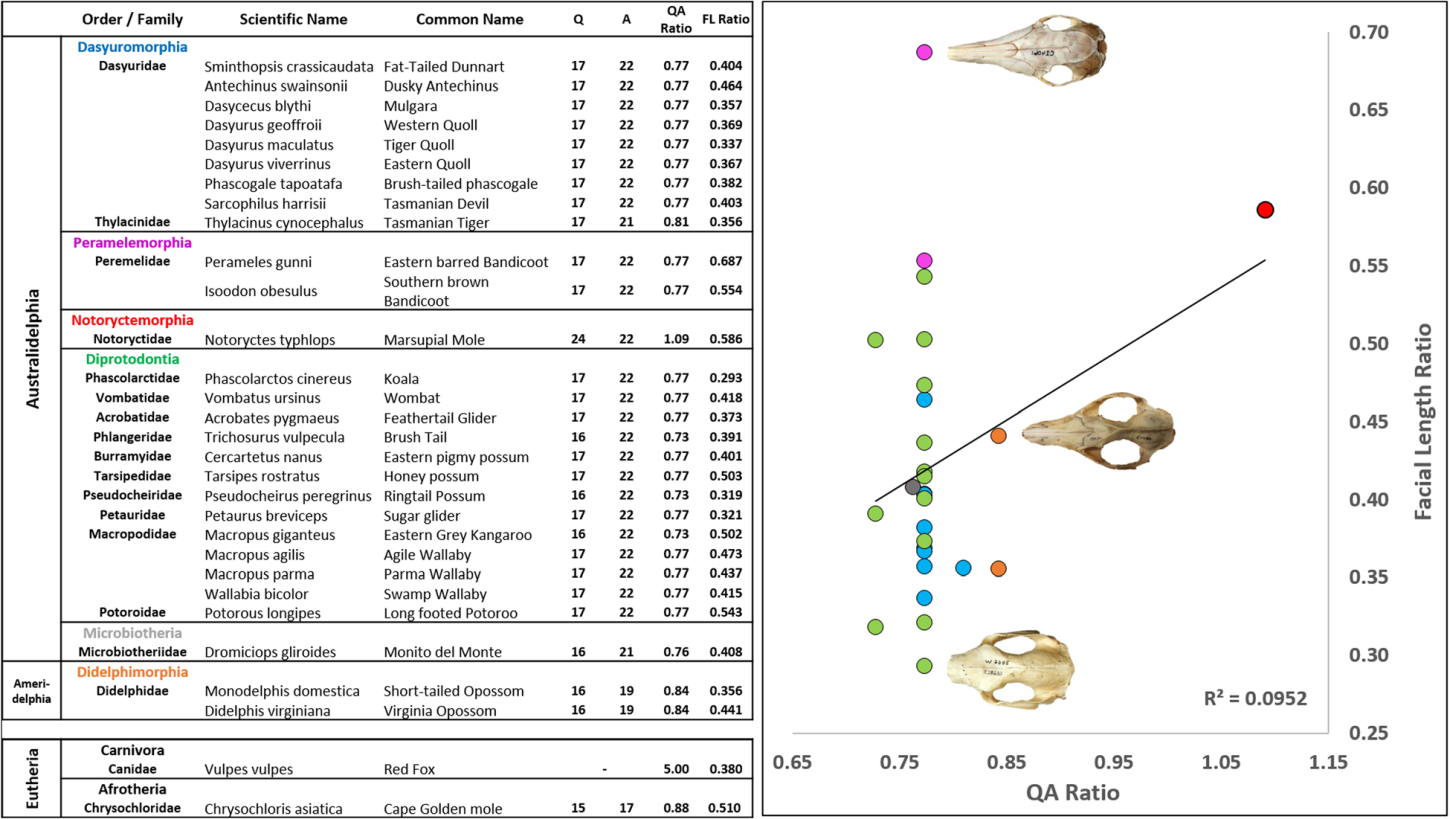
RUNX2 repeat length vs facial length ratio in marsupialsTable (*left panel*) showing the number of Q and A repeats, QA ratio and facial length (FL) ratio across the species examined, grouped into specific orders. The RUNX2 repeat ratio shows little to no correlation with facial length ratio across marsupials at multiple taxonomic levels. Marsupials possess a highly-conserved repeat length of 17Q:22A, especially within the australidelphians. The American marsupials displayed a slightly shorter repeat and the marsupial mole possesses a greatly extended glutamine repeat. Graph (*right panel*) showing facial length ratios plotted against QA ratio for all species shown in the table. *Coloured dots* indicate specific orders as shown in the table on left. When compared to the various spectra of facial length ratios there was no clear correlation (R^2^ = 0.09). Skulls from the Eastern barred bandicoot (*top*), Virginian opossum (*middle*) and koala (*bottom*) are shown to demonstrate variation in craniofacial morphology

**Fig. 5:**
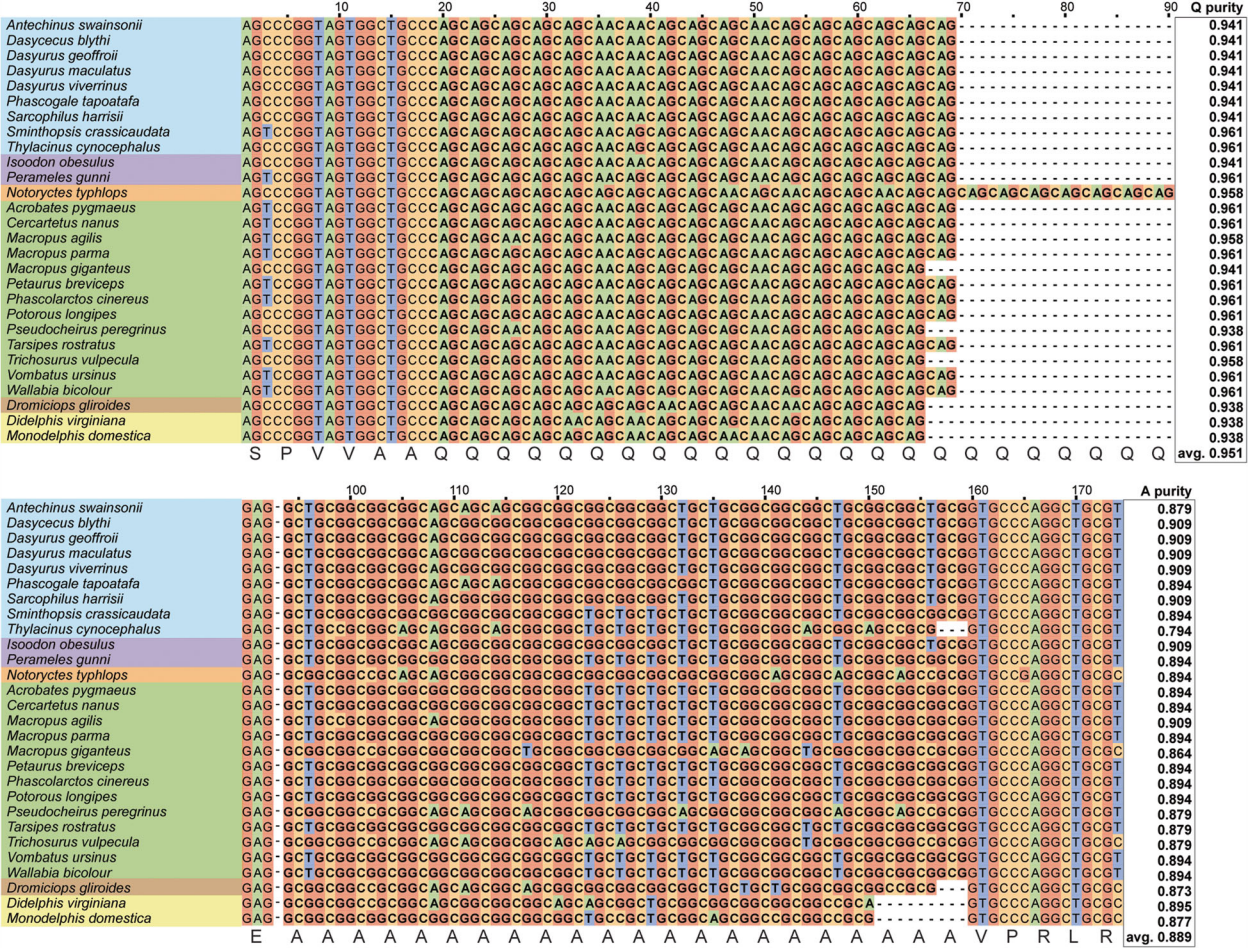
Nucleotide alignment and purity of the RUNX2 repeatAlignment of the RUNX2 repeat with flanking sequence of the 28 marsupials included in this study. Taxa have been colored based on their phylogenetic order. Marsupials display high conservation of the *RUNX2* QA repeat and possess little to no diversity in length except for the marsupial mole (*Notoryctes typhlops*). Marsupials also display a high repeat purity in their glutamine (Q) and alanine (A) codons

We determined *RUNX2* repeat ratios across a broad range of marsupials (Fig. 2) and found that remarkably there was little-to-no variation between individuals at the genus, family and order level (Figs. 4 and 5). The majority of marsupials, specifically australidelphians within the orders Peramelemorphia, Dasyuromorphia and Diprotodontia, show a highly-conserved repeat length consisting of 17Q:22A (ratio 0.77). Slight variation was seen in some members of the order Diprotodontia with one less glutamine (16Q, ratio 0.73) and in the Thylacine (order Dasyuromorphia) with one less alanine (21A, ratio 0.81). The only South American residing australidelphian, the monito del monte (order Microbiotheria), had a slightly shortened 16Q:19A repeat (ratio 0.76), whilst the true South American marsupials, the ameridelphian opossums (order Didelphimorphia) displayed the shortest marsupial repeat with 16Q:18A (ratio 0.84). The largest variation in repeat length was seen in the phenotypically unique marsupial mole (order Notoryctemorphia) with an extended glutamine tract (25Q) making it the only marsupial with a QA ratio above 1 (1.09).

### The *RUNX2* repeat is not correlated with facial length in marsupials

We found that the RUNX2 repeat is not correlated with facial length variation within orders or across the marsupials (r^2^ = 0.09; Fig. 4) due to its high conservation. In addition, we found no similarities in repeat length or composition between the thylacine (0.81) and the red fox (5.0, [14], nor between the marsupial mole (1.09) and golden mole (0.88) despite their remarkable similarities in skull morphology. These results suggest that the large degree of conservation of the *RUNX2* repeat in the marsupials cannot explain their facial length diversity.

### Marsupials display high repeat purity

Given the high level of conservation of the length of the marsupial *RUNX2* repeat, we next explored the codon heterogeneity of the repeat to determine whether marsupials have evolved interruptions to their codon purity as a potential mechanism repressing repeat length variation. We found that members of the various marsupial orders possess high homology in both the repeat and flanking nucleotide sequence of *RUNX2,* yet display specific polymorphisms increasing with phylogenetic distance (Fig. 5)*.* Not only this, but marsupials have maintained a high level of codon purity, despite displaying almost no repeat length variability. We calculated repeat purity using a previously described metric of the number of perfect nucleotide matches in the canonical repeat unit divided by the total length of the repeat [6, 28]. We found that marsupials possess a high purity glutamine repeat (2 possible codons) ranging from 0.93–0.96, whilst the alanine repeat (4 possible codons) ranged from 0.86–0.91, with the exception of the thylacine (0.79) (Figure 5). Both repeat purities were well above the theoretical minima [28] and were in line with those recorded in the placentals.

## Discussion

RUNX2 repeat length variation has been repeatedly correlated with craniofacial length within placental orders [6, 14, 23]. Here we show that this relationship does not hold true in marsupials. Surprisingly, we observed almost no variation in the RUNX2 QA repeat across the entire infraclass, preventing it from acting as a major driver of craniofacial diversity in metatherian marsupials.

Marsupials display a wide range of facial length ratios, similar to that recorded in the placentals (Fig. 1m) [6, 14, 23, 24]. Remarkably, after sequencing the *RUNX2* repeat from 28 marsupial species, it was found that repeat length and composition was extremely conserved, with 20 of the 28 species examined having an invariant RUNX2 repeat of 17Q:22A (ratio 0.77) (Fig. 4). The two sampled opossums (*Monodelphis domestica* and *Didelphis virginiana*, order Didelphimorphia), showed a slightly shortened repeat consisting of 16Q:19A (ratio 0.84). The Didelphimorphia are derived from one of the most basal marsupial and South American residing lineages [26] and are perhaps representative of the ancestral marsupial repeat before the divergence of the australidelphian marsupials. The monito del monte (*Dromiciops gliroides,* order Microbiotheria), the only South American residing australidelphian marsupial [29], has a repeat length that is intermediate to the South American and Australian marsupials, comprised of 16Q:21A (ratio 0.76). Species from the three orders that make up the majority of Australian marsupials (order Dasyuromorphia, Peramelemorphia and Diprotodontia) predominantly showed a conserved 17Q:22A allele (with 3 members showing 16Q:22A and the thylacine reconstruction showing 17Q:21A). It is worth noting that the koala (*Phascolarctos cinereus,* order Diprotodontia) and the Eastern barred bandicoot (*Perameles gunnii,* order Peramelemorphia) showed no difference in their RUNX2 repeat length, despite occupying opposite ends of the facial length spectrum in marsupials (Figure 4). The one outlier with respect to RUNX2 repeat ratio was the marsupial mole (*Notoryctes typhlops*, order Notoryctemorphia) which has an extended glutamine tract (24Q:22A, ratio 1.09). Whilst the Notoryctemorphia is classed within the Australidelphia, its true phylogenetic position is still disputed [30]. Marsupial moles are highly specialized burrowers and perhaps have been under their own unique and extreme selective pressures to arrive at their highly unusual morphology and marsupial RUNX2 repeat composition. Even when the marsupial mole RUNX2 repeat ratio is considered, the range of variation seen in marsupials (0.76–1.09) is minimal compared to that seen in the Primates (1.1–1.7 [23]), the Carnivora (1.2–5.33 [6,14]) and across placentals as a whole (0.82–5.33 [24]). As expected, despite their extraordinary morphological convergence and similarities in craniofacial morphology, the repeat lengths of the thylacine and red fox and the marsupial and placental moles were vastly different, likely owing to their distant divergence and genomic background.

The extreme conservation in RUNX2 QA repeat length seen across marsupials was unexpected due to the typical volatility of such sequences [18, 20] and the large amount of inter-specific and intra-specific variability seen across the placentals [6, 14, 23, 24]. While each of the *RUNX2* repeats sequenced in this study were from a single individual, the extremely low levels of variation between individuals at the genus, family and order level suggests it is highly unlikely that marsupials would possess any intra-species variability. Therefore, we hypothesised that marsupials must have evolved variation in the codon usage of the QA repeat as a repressive mechanism to control replication slippage. The longer and purer the repeat, the more unstable and prone to replication error [18, 19]. However, our results show that the marsupial polyglutamine, polyalanine repeat tract is of a high repeat purity (0.95 & 0.89) despite showing almost no length variation. The average marsupial *RUNX2* glutamine repeat purity (0.95) was observed to be consistent with that observed in dogs (0.96) [6], Primates (0.94) [23]) and throughout other linages of placental mammals (0.94) (calculated from [22]). The marsupial alanine repeat purity (0.89) is also similar to that found in humans (0.88), Primates (0.90) and other placental mammals (0.90), but lower than that observed in domestic dogs (0.95) [6, 23, 24]. Whilst these values only represent purity at a single locus, they are also in line with observed genomic repeat purity in the various families of the Carnivora [28]. Given that both the marsupials and placentals show highly similar *RUNX2* repeat purities, yet the marsupials possess little to no length variation, our data suggests that marsupials must have evolved other mechanisms to repress RUNX2 repeat mutation and slippage events as a means to maintain repeat length integrity.

The conservation of the *RUNX2* repeat length in marsupials suggests that there is strong evolutionary constraint on its function. Marsupial development is unique in that the young are born in a highly altricial state, yet display accelerated anterior skeletal development [1, 31, 32]. Marsupial neonates display accelerated development and ossification of the shoulder girdle and forelimbs for climbing into the pouch [31, 32]. In addition, they require accelerated development of the facial skeleton, particularly ossification of the upper and lower jaw, to enable attachment to the teat and sucking [33, 34]. Owing to this mode of development it has been hypothesized that marsupials are subjected to strong developmental constraints, resulting in limitations to their ontogenic flexibility in skeletal development and a severe reduction to their adult craniofacial morphological diversity [1, 31, 32, 35]. Given that *RUNX2* is a major regulator of osteogenesis and skeletal development, any major alterations of the repeat leading to changes in its expression and transactivation [15–17] or timing of ossification of the core skeletal elements may be pleiotropic and negatively selected against. We suggest that purifying selection has maintained the *RUNX2* repeat throughout marsupial evolution as a mechanism to maintain their constrained skeletal development.

## Conclusions

Together our data have shown that *RUNX2* repeat length is highly conserved across the marsupials both in repeat length and sequence composition. These data discount *RUNX2* QA repeat length variation as a driver of craniofacial diversity in marsupials unlike in placental mammals. Furthermore, *RUNX2* repeat length is not correlated with convergent phenotypes between placental and marsupial species. Therefore, marsupials must have evolved other mechanisms to drive facial length diversity. The unprecedented degree of repeat conservation across the marsupials implies strong purifying selection to maintain a stable allele. We propose that the extreme conservation in repeat length is constrained by the early ossification of the anterior skeleton to enable climbing and suckling of the altricial marsupial young.

## Methods

### Species sampling

We sampled marsupial species occupying a range of ecological niches and specialised diets with a diverse array of craniofacial morphologies (Figs. 1, 2). The taxa included representatives from each of the marsupial orders, with the exception of the South American Paucituberculata due to sample availability. Marsupial tissues were collected from various sources (Additional file 1: Table S1) and genomic DNA was extracted using the DNeasy Blood & Tissue kit (QIAGEN). For each species included in the study, we sourced representative skulls to record facial length measurements (Additional file 2: Table S2). Skulls were obtained from the Museum Victoria Mammalogy collection. For rarer samples, we further acquired 3D CT cranial data from DigiMorph (University of Texas; http://www.digimorph.org) online skull depository.

#### Facial length measurements

Facial length measurement parameters were adapted from previous studies [6, 14, 24]. We measured facial length and total skull length, as a proxy of size, on the right and left side of the skull in male and female specimens, unless otherwise noted (Additional file 2: Table S2). Facial length was measured as the distance between the juncture of the jugal, lacrimal and maxillary bones, and the anterior juncture of the nasal and premaxilla. The skull size proxy was the measurement of the anterior juncture of the premaxilla and maxilla to the lateral edge of the occipital condyle (Fig. 3). Facial length was divided by the skull proxy and averaged for both sides, then averaged between the sexes to gain a sex-adjusted, facial length ratio (Additional file 2: Table S2). The range of marsupial facial length ratios were compared with previously published placental [24] and carnivoran [14] datasets by observing the distribution of ratios through the generation of a box and whisker plot (Fig. 1m).

### *RUNX2* repeat amplification and analysis

The *RUNX2* repeat was amplified in triplicate from genomic DNA from each of our specimens using marsupial-specific *RUNX2 QA* repeat flanking primers [5′-ATCCGAGCACCAGTCGGCGGTTCAG-3′, 5′-GTGGTCAGCGATGATTTCCAC-3′] based on previous studies [6, 14, 24]. To minimize PCR error, the *RUNX2* repeat was amplified using Phusion Hot Start II High Fidelity Polymerase (Thermo Scientific). The resulting PCR products were purified (QIAquick Gel Extraction Kit, QIAGEN), and sequenced on a Capillary electrophoresis sequencer (Applied Biosystems) (Centre for Translational Pathology, University of Melbourne). As a positive control for accuracy, we amplified the human and mouse *RUNX2* repeat and compared against publicly available sequence data (not shown). For the convergent repeat analysis, we retrieved the sequence for the Golden Mole (*Chrysochloris asiatica)* from GenBank*,* [XM_006860524.1.]

#### Sequence alignment

The obtained PCR sequence trace files (~200 bp) were imported into ChromasPro2.0.1 (Technelysium Pty Ltd.) for quality trimming and nucleotide base calling. Triplicate traces were obtained for each species and used to generate a consensus sequence.

#### Thylacine repeat reconstruction

Thylacine *RUNX2* orthologous sequences were obtained from raw whole genome shotgun Illumina and Proton reads and assembled using ChromasPro2.0.1. Additional *RUNX2* sequence was PCR amplified using Dasyuromorphia specific primers immediately flanking the repeat to amplify a 180 bp repeat-containing fragment [5′-GACGTGAGCCCGGTAGTG-3′, 5′-CATGGTGCGATTGTCGTG-3′]. The resulting PCR products were Sanger sequenced in both directions, and aligned against the NGS reads. The thylacine *RUNX2* repeat consensus sequence was then generated from the aligned sequence data.

#### Statistical analysis

We compared marsupial RUNX2 repeat ratios with facial length ratios across the sampled marsupial taxa and generated a linear regression line and calculated the coefficient of determination (R^2^) using excel [6, 14, 23].

#### Repeat length determination and purity

To determine the RUNX2 repeat length and QA ratio, the resulting 28 marsupial nucleotide sequences were aligned and translated in MEGA7 [36]. The RUNX2 QA repeat ratio was then calculated by dividing the number of consecutive glutamines by consecutive alanines. Glutamine and alanine tandem repeat purity was calculated from each species’ nucleotide consensus based on previously described methods [6, 28]. The resulting marsupial repeat purity values were compared to those reported for domestic dogs and other carnivorans [6, 28], and to purity values calculated from publicly available sequence data for the Primates [23] and other placental mammals [24] (Additional file 3: Table S3).

## Additional files

Additional file 1: Table S1. List of marsupial tissues and DNA samples used. (XLSX 11 kb)

Additional file 2: Table S2. Facial length measurement data. (XLSX 20 kb)

Additional file 3: Table S3. Repeat purity data. (XLSX 26 kb)

Additional file 4: Table S4. Copywrite information for images used in the study. (XLSX 12 kb)

## Abbreviations

3D CT: three-dimensional computed tomography
CCD: cleidocranial dysplasia
PCR: polymerase chain reaction
QA ratio: ratio of glutamines to alanines
RUNX2: Runt-related transcription factor 2
